# Practical Use of Nitrous Oxide

**Published:** 1904-07-15

**Authors:** E. R. Jackson

**Affiliations:** Grand Rapids, Mich.


### PRACTICAL USE OF NITROUS OXIDE.

BY E. R. JACKSON, D.D.S., GRAND RAPIDS, MICH.

As nitrous oxide, in the hands of a few, proves to be a satisfactory agent for producing anesthesia, yet attempted and discarded as objectional by many, it would seem a worthy subject for consideration. I have not made its administration a special study, but have administered nitrous oxide frequently in my practice during the past twenty years, and believe I have seen nearly all its commendable as well as objectionable manifestations. I have used it with patients of all ages, from three to eighty-five years, and with people representing nearly every condition of mind and body, and I have never lost a patient, nor have I seen a single case showing dangerous symptoms which I now believe to have been directly caused by the anesthetic, or that might not have been more favorable with a different anesthetic. Ought we to be surprised, or should we denounce the anesthetic, because a nervous woman is troubled with hysteria after operating upon her in the excitant stage of nitrous oxide anesthesia? Where should we place the fault if our patient screams and fights for liberty, when we attempt to operate while the sensory nerves are still susceptible to pain? Should you condemn the anesthetic which, if carried to the proper stage, is sure to protect you and your patient from such unpleasantness? Would it not be better to believe ourselves capable of error, and by the exercise of a little common sense, try to do what others have done with the same agent?

The principal objection to nitrous oxide, as generally expressed, is brevity of anesthesia, and this must be admitted. Yet, if we improve the time as we may, there will seldom be reason to complain of lack of time, as most operations will have been completed before the patient returns to sensibility.

Nitrous oxide, most frequently used in extracting, may also be employed to great advantage in any operation where pain and nervousness makes a patient uncontrollable; excising teeth for crowns, pulp extirpation, replanting, cutting forms in very sensitive teeth for filling, lancing in abscessed conditions, or any operation the performance of which requires not more than a minute of time. If you would succeed in any operation under nitrous oxide anesthesia, you must first ascertain just what work you have to do. Examine with the view of forming a diagram of the case in your mind, as it appears in the patient's *mouth*, and *never* proceed with the anesthetic until this is acquired. Without a perfect understanding of the case confusion will prevent accuracy, and will so much retard the speed of the work that the duration of anesthesia will have elapsed before the work is completed. You should not expect to operate skillfully under personal excitement, and coolness is possible only where there is perfect understanding. Select suitable instruments for the performance of the work, decide where it is best to begin, and place the instruments in position on the bracket table, so that you can reach them in their turn, as you have decided to use them. This will help to avoid unnecessary change of instruments, and consequent waste of time. Get an idea of the temperament and general condition of your patient without arousing his suspicion of your anxiety, and while it is well to show interest in his safety, and some regard for his nervousness, I think it unwise to question him extensively or convey to him the idea that much depends upon the heart's action. What knowledge you gain is overbalanced by his increased nervousness. He fights you and the anesthetic at the first flutter of the heart, because the importance of this has been impressed upon him.

I think it is well to tell every patient something of the unpleasantness which he is sure to experience, assuring him that this need not alarm him. Advise him to take full, quick breaths, and to exhale forcibly, the inhalations and exhalations will be more uniform and the liability of cessation of respiration will be lessened.

After beginning the anesthetic say nothing, and allow no one else to speak to the patient nor to you. He cannot understand clearly, and his effort to retain consciousness retards anesthesia and exhausts the patient. Never advise a patient to hold the hand up, to move a finger, nor anything that will require an effort on his part, or give him an idea that you depend upon him to inform you as to the stage of anesthesia. You cannot safely rely upon such proofs, and must decide by the sound of breathing, and the absence of sensitiveness of the conjunctiva long after he would be unable to move a finger. This is the most trying moment for the operator. Upon your management depends your success and your patient's safety. You are about to begin the work the success of which is not certain, and the result of your effort can only be known when you have finished. To carry the anesthesia to the point where the best result is possible, at the risk of a human life given to you through confidence to protect, is by no means a nerve tonic; and to lose sight of the fact that there is always danger at this time would breed carelessness, which has no place in this work.

With nearly all patients it is safe to carry the anesthesia to a degree where the muscles relax; but this requires the most careful attention to the patient's condition, and is not advocated by any one whom I have known to discuss this subject. It is generally believed to be unsafe. If the patient has been allowed a breath of air occasionally, during the administration, and symptoms of lividity are absent and the respiration uniform, I believe it far better to continue to this point than to attempt the work while the muscles are rigid. I learned this in the hospitals where for surgical work in one case I continued the anesthesia over a period of thirty-five minutes, in which I was compelled to *keep* the muscles relaxed. I have administered nitrous oxide in a hospital in Grand Rapids for surgical work on several occasions with perfect success, and the patients were in most cases revived within a minute after the work was completed.

In all ordinary cases, I use nothing but cold water in the face to revive a patient, yet it is always well to have within your reach proper resuscitants. A hypodermic syringe containing $\frac{1}{50}$ of strychnia, a respiratory excitant or a cardiac stimulant, as amyl nitrite, nerve quieting remedies, bromide of potassium, etc., and to understand and employ without delay the proper methods for artificial respiration in case of cessation. Always use a mouth prop unless the teeth are all removed from one of the maxillaries. It is especially convenient in events where the tongue, after anesthetized, is drawn back over the glottis and closing the air passages so that it becomes necessary to thrust the finger into the throat to recover it. A pair of tongue forceps should be kept conveniently by for such emergencies.

I never administer nitrous oxide with the clothing tight about the body or neck of the patient, nor except in the presence of an assistant or third party. Use nothing but liquified gas. Test the apparatus to see that it is in good working order immediately before using. Keep cool and remember what you have to do.

